# Constructing a Predictive Model for STH and Schistosomiasis Classification From Microscopic Images

**DOI:** 10.1155/bmri/8074581

**Published:** 2025-11-29

**Authors:** Etefa Belachew, Kris Calpotura, Abrham Adamu, Berhanu Getachew

**Affiliations:** ^1^ Faculty of Electrical and Computer Engineering, Jimma University–Institute of Technology, Jimma, Ethiopia; ^2^ School of Electrical and Computer Engineering, Haramaya University, Harar, Ethiopia, haramaya.edu.et; ^3^ Ethiopian Public Health Institute, Addis Ababa, Ethiopia, ephi.gov.et

**Keywords:** deep learning, digital image processing, machine learning, pretrained models, STH and schistosomiasis, ViT

## Abstract

Soil‐transmitted helminths (STHs) and schistosomiasis are widespread parasitic diseases in tropical regions, particularly in Africa, with substantial health and socioeconomic burdens. Early diagnosis and treatment are critical for mitigating these impacts. Conventional microscopy‐based diagnosis was time‐consuming and labor‐intensive, posing challenges in resource‐limited settings such as Ethiopia. This study developed an innovative system that combined machine learning (ML) and deep learning to analyze microscope images of parasite eggs, improving diagnostic speed and accuracy compared to traditional CNN‐only approaches. We compared a hybrid CNN–ML approach with standalone deep learning models and vision transformers (ViTs) for classifying five categories: *Ascaris*, hookworm, schistosomiasis, *Trichuris*, and negative samples. The dataset comprised 1490 images from the Ethiopian Public Health Institute, processed with resizing, normalization, and augmentation. CNN architectures (VGG16, ResNet50, DenseNet121, MobileNetV2, and EfficientNetB0) and ViT served as feature extractors, with ML classifiers (SVM, XGBoost, KNN, RF, and DT) performing the predictions. The hybrid CNN–ML model outperformed standalone models, with VGG16‐SVM and VGG16‐XGBoost achieving the highest test accuracy of 99.31% and 99.35%, respectively. In contrast, standalone CNNs showed lower accuracy (VGG16: 79.98%; DenseNet121: 84.12%). Negative samples were classified with high accuracy across models, while parasite classes exhibited varying performance depending on the architecture. This system enhances diagnostic utility in low‐resource settings by enabling real‐time analysis. However, limitations include a small, long‐stored dataset with limited diversity and potential degradation, which may affect model generalizability.

## 1. Introduction

Schistosomiasis and soil‐transmitted helminths (STHs) are among the most widespread neglected tropical diseases (NTDs), collectively affecting more than 1.5 billion people, particularly in sub‐Saharan Africa, Asia, and Latin America [1, 2]. However, these diseases are not confined to tropical and subtropical regions. Emerging evidence shows endemic transmission of schistosomiasis in parts of Europe, including Corsica, France, and the Balkans, raising global concerns about its re‐emergence in nonendemic areas [3]. With increasing international travel and migration, both schistosomiasis and STHs are becoming relevant even in high‐income countries with historically low prevalence.

These parasitic infections are caused by distinct organisms with different transmission routes and clinical profiles. STHs are intestinal worms primarily acquired through ingestion or skin penetration via contact with contaminated soil, water, or food. The most common STHs affecting humans include *Ascaris lumbricoides* (roundworm), *Trichuris trichiura* (whipworm), and hookworms (*Necator americanus* and *Ancylostoma duodenale*) [4]. In contrast, schistosomiasis is caused by trematodes of the genus *Schistosoma*, primarily *S. mansoni* and *S. haematobium*, which infect hosts through skin contact with freshwater contaminated by cercariae released by infected snails [3].

In Ethiopia, the World Health Organization (WHO) has identified 251 districts endemic for STHs and 67 for schistosomiasis. Accordingly, approximately 18 million and 14 million school‐aged children require mass drug administration (MDA) for STHs and schistosomiasis, respectively [5]. This highlights the urgent need for scalable, efficient, and reliable diagnostic solutions to support control and elimination programs.

Traditional diagnosis of these infections primarily relies on stool microscopy. For STHs and schistosomiasis (*S. mansoni*), the Kato–Katz thick smear technique is widely used to identify parasite eggs in fecal samples [6]. In contrast, *S. haematobium* is typically diagnosed by urine filtration and microscopy, not stool examination, with peak egg excretion occurring around midday [3]. These diagnostic methods, although considered gold standards, are resource‐intensive, time‐consuming, and dependent on skilled personnel. Their performance declines in low‐intensity infections and field settings, where proper sample preparation and experienced technicians may be lacking [7].

Manual microscopy is also susceptible to observer variability and interpretation bias. Human error—particularly in low‐prevalence settings—can lead to false positives, especially when artifacts are misidentified as eggs. Studies have shown interobserver discrepancies and a drop in diagnostic accuracy with fatigue or when examining poor‐quality samples [7, 8]. For example, Bosch et al. [7] showed that factors such as storage time and sample handling significantly affect egg counts, contributing to misclassification. These limitations stress the need for automation and standardization [9].

In response, deep learning (DL) and ML approaches have emerged as promising alternatives for parasite detection. Convolutional neural network (CNN)‐based methods can extract intricate image features for automatic classification. Studies have demonstrated the utility of these models in identifying parasite eggs, mapping disease prevalence, and overcoming human limitations. For instance, Scavuzzo et al. [10] applied XGBoost with SHAP (Shapley additive explanation) to predict STH risk. Parra et al. [11] used MobileNet for classifying six parasite types with 94.26% accuracy, outperforming custom CNNs. Similarly, Lee et al. [12] developed the helminth egg analysis platform (HEAP), integrating Single‐Shot MultiBox Detector (SSD) and Faster R‐CNN to improve diagnostic throughput. Another study applied DL on whole‐slide images (WSIs) for light‐intensity infections, achieving up to 92% sensitivity and 98% specificity [13].

Despite these advances, standalone CNN models often struggle with overfitting, particularly when trained on small or imbalanced datasets. To mitigate this, hybrid architectures combining CNN‐based feature extraction with classical ML classifiers like XGBoost, support vector machine (SVM), random forest (RF), and DT have proven effective. These classifiers handle limited datasets better and define clearer decision boundaries [14]. Additionally, classical ML models are generally more interpretable and resilient to noise.

This study also evaluates vision transformer (ViT), a state‐of‐the‐art architecture leveraging self‐attention mechanisms originally developed for natural language processing. Unlike CNNs, ViT segments images into patches and processes them like tokens, capturing both local and global dependencies [15]. This enables ViT to model complex spatial relationships, which is critical in medical imaging.

DenseNet121 and VGG16 were specifically chosen due to their success in biomedical applications. DenseNet121 facilitates feature reuse and efficient gradient propagation, making it suitable for training on small datasets [16]. VGG16, known for its simplicity and depth, provides a solid baseline for comparison due to its consistent performance across various image classification tasks [17].

In this study, we aim to classify five parasite categories—*Ascaris*, *Trichuris*, hookworms, *Schistosoma*, and negative samples—using predictive models built from CNN feature extractors and classical machine learning classifiers. We also compare the performance of standalone CNN and ViT classifiers. Our goal is to enhance diagnostic automation in low‐resource settings such as Ethiopia, where conventional infrastructure is limited, but the burden of parasitic disease remains high.

## 2. Methods

This study adopts an experimental modeling framework to classify STHs and schistosomiasis from microscopy images. Two main approaches were developed: (1) DL models, including CNNs and ViT, and (2) hybrid models integrating CNN‐based feature extractors with ML classifiers. A comparative evaluation was conducted across five parasitic classes: *Ascaris lumbricoides*, *Trichuris trichiura*, hookworm species (*Necator americanus*/*Ancylostoma duodenale*), *Schistosoma* spp., and negative samples.

### 2.1. System Architecture

**Figure 1:**
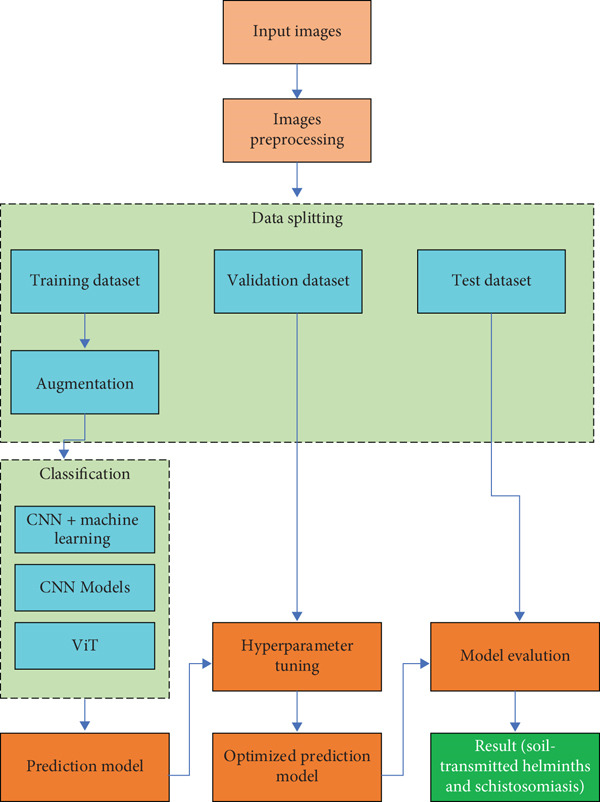
High‐level classification pipeline for soil‐transmitted helminths (STH) and schistosomiasis. The workflow includes image acquisition, preprocessing, dataset splitting, augmentation, classification (CNN + ML hybrids, CNNs, and ViTs), hyperparameter tuning, and model evaluation. The optimized model generates final classification results.

**Figure 2:**
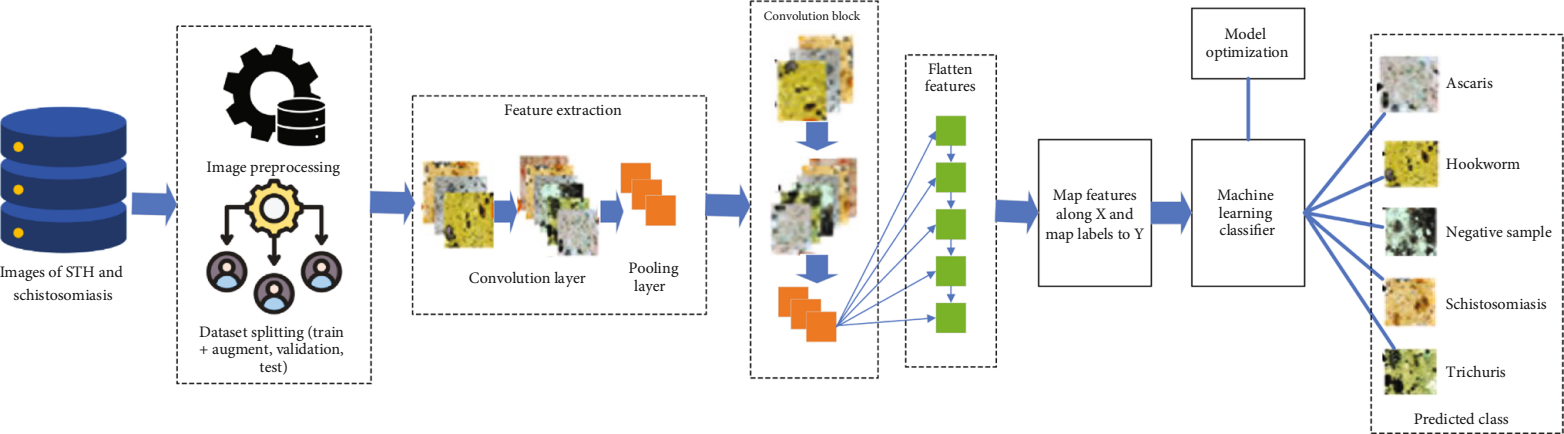
Detailed CNN + machine learning hybrid architecture for classifying STH and schistosomiasis. Images undergo preprocessing and dataset splitting, followed by feature extraction through convolution and pooling layers. Features are flattened, mapped to labels, and classified using machine learning algorithms. Model optimization yields predictions into one of five categories: *Ascaris*, Hookworm, *Trichuris*, schistosomiasis, or negative.

The proposed framework for classifying STHs and schistosomiasis follows a structured pipeline that integrates image preprocessing, feature extraction, model training, and evaluation. This methodology is illustrated at two levels of detail: a high‐level overview (Figure 1) and a detailed CNN‐based hybrid architecture (Figure 2).

The process begins with the acquisition of microscopic images containing STH and schistosomiasis specimens. These images are carefully annotated by parasitology specialists to ensure high‐quality ground truth labels. Accurate annotation is critical for supervised learning and ensures that the models are trained on reliable and representative data.

The images undergo preprocessing, which involves resizing to a uniform resolution, pixel value normalization to a range between 0 and 1, and, where necessary, noise reduction and contrast enhancement. Following preprocessing, the dataset is divided into training, validation, and test subsets. The training set is expanded using data augmentation techniques (flipping, rotation, contrast adjustment, zooming, and shifting) to improve generalization and reduce overfitting.

At the classification stage, multiple modeling strategies are employed (Figure 1). These include

1. CNN + machine learning hybrids, where CNNs act as feature extractors and classical classifiers such as SVMs or RFs are applied to the extracted feature vectors.
2. Standalone CNN models, which directly learn hierarchical image representations for classification.
3. ViT models, which divide images into patches and capture global dependencies through self‐attention mechanisms.

The detailed CNN + ML hybrid workflow is presented in Figure 2. Here, convolutional layers apply filters to extract local morphological features of parasite eggs, while pooling layers reduce dimensionality and retain salient information. Stacked convolutional blocks progressively learn more abstract features, which are then flattened into one‐dimensional vectors. These feature vectors are mapped along the input space *X*, with corresponding class labels (*Y*) representing *Ascaris*, hookworm, *Trichuris*, schistosomiasis, or negative (nonparasitic) samples. A machine learning classifier then performs the final prediction task, with hyperparameter optimization ensuring optimal accuracy and generalization.

The model evaluation phase involves testing the optimized models on unseen data. Performance metrics such as accuracy, precision, recall, and *F*1‐score are calculated to provide a comprehensive assessment. The best‐performing model is identified as the optimized prediction model and produces automated classification results that support accurate diagnosis of STH and schistosomiasis.

### 2.2. Data Collection and Sample Preparation

#### 2.2.1. Source and Location

The dataset was systematically collected through a collaborative effort between researchers and the Ethiopian Public Health Institute (EPHI), ensuring adherence to standardized diagnostic protocols. Microscopy slides were obtained from two well‐equipped laboratory facilities: the EPHI National Laboratory Center in Addis Ababa, which serves as Ethiopia′s primary reference lab for infectious diseases, and the pathology laboratory at Jimma University, a leading institution in medical research and regional healthcare.

#### 2.2.2. Sample Collection and Labeling

The researcher carefully collected and labeled the data. The crucial next step was to prepare it in a structured manner by organizing it into folders corresponding to specific classes. Microscopic images of STH and schistosomiasis eggs were collected from primary sources, including the EPHI, clinics, and research laboratories. These images were captured using high‐resolution microscopes and standardized imaging protocols. They were then preprocessed through resizing, normalization, and augmentation. The data was organized into five classes—*Trichuris*, *Schistosoma*, negative, hookworm, and *Ascaris*—within their respective folders. Finally, the dataset was split into training, validation, and test sets to facilitate accurate model development.

Experts from EPHI and Jimma University manually labeled STH and schistosomiasis image datasets, enhancing them with metadata. Collaborating with EPHI, data was collected from high‐prevalence Ethiopian regions using the Kato–Katz technique, resulting in 285 meticulously prepared and labeled smear slides.

The Kato–Katz technique, a WHO‐recommended quantitative method for diagnosing STH, involves preparing standardized fecal smears (41.7 mg) cleared with glycerin‐coated cellophane. Eggs are microscopically counted to determine infection intensity (eggs per gram). While it is cost‐effective and field‐friendly, its sensitivity depends on operator skill and egg stability. To ensure accuracy, duplicate smears were analyzed, and two microscopists independently reviewed each slide, with a third expert resolving discrepancies. This rigorous protocol enhanced dataset reliability for predictive modeling [3, 6, 18].

A total of 1490 high‐quality images were captured at 40× magnification from two laboratory sites, with 300 images per class to avoid bias, creating a robust and representative dataset for model training. The details are summarized in Table 1.

**Table 1:** Datasets collated from the Ethiopian Public Health Institute. As shown in the table, the dataset consists of 1490 microscopic images collected from 285 slides distributed among five parasite categories and negative samples.

**Class**	**No. of slides**	**Image taken**	**Format**
*Trichuris trichiura*	67	300	.Jpeg.bmp.jpg
*Ascaris*	52	300	.Jpeg.bmp.jpg
Negative samples	53	290	.Jpeg.bmp.jpg
Schistosomiasis	66	300	.Jpeg.bmp.jpg
Hookworm	47	300	.Jpeg.bmp.jpg
Total	285	1490	.Jpeg.bmp.jpg

Utilizing JPEG and BMP formats offers distinct advantages for image preprocessing and machine learning. JPEG′s compression capability optimizes storage space and facilitates the rapid loading of large datasets. In contrast, BMP employs lossless compression, such as run‐length encoding, preserving fine pixel resolution essential for generating high‐quality images. Despite their differences, both formats guarantee compatibility with leading machine learning libraries. Their efficient decoding accelerates preprocessing tasks like resizing and augmentation. Ultimately, JPEG provides a versatile compression technique that balances quality and file size for efficient and streamlined data handling in machine learning workflows.

#### 2.2.3. Ambiguous and Negative Samples

Negative slides were carefully screened to confirm the absence of eggs. Slides with ambiguous or unclear visual features were excluded to avoid false labeling and confounding during training.

#### 2.2.4. Sample Size Justification

The dataset comprises 1490 microscopy images derived from 285 Kato–Katz stool slides (≈300 images per parasitic class; negative = 290). Although this number is modest for training deep networks from scratch, we used transfer learning with pretrained CNN backbones, extensive image augmentation, hybrid CNN → ML classifiers, and stratified cross‐validation to increase sample efficiency and reduce overfitting risk. Practical power/precision calculations further support our design: A basic two‐proportion power calculation indicates that ≈76 images per group are sufficient to detect a large accuracy improvement from 80% to 95% (*α* = 0.05, power = 0.80), and the sample size required to estimate an observed accuracy of ≈99.35% with ±1% precision (95% CI) is ≈249 images. Because each class contains ≈300 images, per‐class sample sizes meet these practical thresholds. We note, however, that multiple images originate from the same slides, which reduces the effective independent sample size; therefore, we split the data by slide during experiments and report both image‐level and slide‐level results. Finally, we acknowledge that external validation on independent slide collections will be required to confirm generalizability.

### 2.3. Image Acquisition and Preprocessing

Image processing is a crucial step in developing DL models for image data, as it leverages the spatial structure of images for effective feature mapping. A key part of this process involves splitting the data into training, validation, and test sets and often includes resizing. Image preprocessing employs various techniques to enhance image quality. This is particularly important for medical images, which frequently contain irrelevant artifacts. These preprocessing techniques are applied prior to classification to remove such irrelevant elements, thereby improving both image visualization and overall model performance [19].

**Figure 3:**
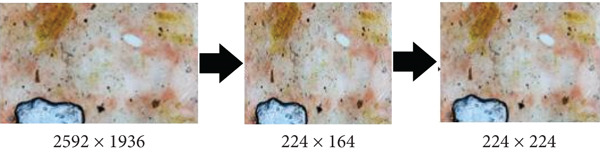
Image resizing using aspect ratio and padding to avoid feature distortion.

To prepare images for model input, the following preprocessing steps were applied:

• Resizing. Images were originally captured at a resolution of 2592 × 1936 and resized to 224 × 224. Preserving the aspect ratio during resizing is crucial, especially during downsampling, to maintain the image′s original proportions. The aspect ratio, defined as the width divided by height, must be preserved to avoid distortions such as stretching or compression, which can impair visual interpretation, particularly in fields like medical imaging where accuracy is vital. To prevent this, resizing was performed by adjusting one dimension and calculating the other proportionally, and when the target size was smaller than the original, padding (adding borders) or cropping (removing parts) was applied to fit the image without distorting its aspect ratio. These techniques ensure that the resized image retains its essential features and proportions for accurate analysis (Figure 3).

• Normalization. Normalization was essential for stable model training, as it converts input data into consistent, smaller values. Unscaled values can slow optimization and cause instability in gradient‐based methods. Scaling RGB pixel intensities to [0,1] facilitates weight updates, accelerates training, enhances accuracy, and improves the overall effectiveness of CNNs.
• Data augmentation. Data augmentation expands datasets to reduce overfitting and improve model generalization. Using ImageDataGenerator, techniques such as rotation, shifting, shearing, zooming, flipping, and fill modes introduce variability. These transformations simulate real‐world conditions, preserve essential features, and enhance robustness, enabling DL models to perform more accurately on unseen data.

These steps increased dataset diversity and minimized overfitting, especially critical due to the limited size of the dataset [20, 21].

### 2.4. Model Architectures and Rationale

#### 2.4.1. CNN Architectures

For feature extraction and classification, we employed five pretrained CNN architectures selected for their proven performance in medical image analysis: VGG16 [17], valued for its straightforward yet effective design in medical imaging tasks; ResNet50 [22], which utilizes residual connections to address vanishing gradient problems in deep networks; DenseNet121 [16], featuring dense interlayer connectivity that enhances feature reuse and model efficiency; MobileNetV2 [23], optimized for lightweight applications and potential mobile deployment; and EfficientNetB0 [24], which achieves optimal accuracy–efficiency trade‐offs through compound scaling. Each architecture was implemented in two configurations: as an end‐to‐end classifier fine‐tuned on our dataset and as a feature extractor for downstream hybrid models.

All CNN backbones (VGG16, ResNet50, DenseNet121, MobileNetV2, and EfficientNetB0) were initialized with ImageNet‐pretrained weights. For the standalone CNN classifiers, the convolutional base was frozen up to the final block, and only the top dense layers were trained; the last convolutional block was unfrozen and fine‐tuned with a low learning rate (1e − 5) to adapt to domain‐specific features while avoiding overfitting. For the hybrid CNN–ML models, the CNNs were used solely as feature extractors: All convolutional layers were frozen, and no fine‐tuning was applied. Features were extracted from the global average pooling (GAP) layer immediately before the fully connected classification head. The resulting feature vectors (e.g., 512 dimensions for VGG16 and 1024 for DenseNet121) were flattened and standardized (*z*‐score normalization) before training the classical machine learning classifiers (SVM, XGBoost, KNN, RF, and decision tree).

#### 2.4.2. ViT

**Figure 4:**
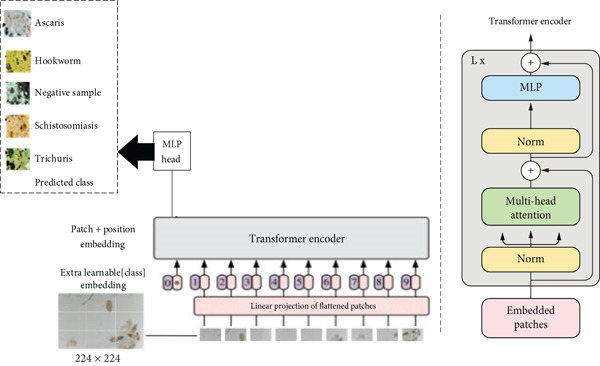
Vision transformer system architecture.

The ViT architecture [15] introduces a novel approach to image analysis by adapting transformer‐based methods traditionally used in natural language processing. The input image is first divided into patches, which are flattened and linearly projected to form embeddings, as shown in Figure 4. Positional embeddings are then added to preserve spatial information. These patches are passed through a transformer encoder composed of multiple layers with multihead attention and multilayer perceptron (MLP) blocks, enabling the model to capture long‐range dependencies. The encoder output is subsequently processed through an MLP head for classifying STH and *Schistosoma*. By focusing on the most relevant image features, the model achieves reliable predictions even when trained on limited data.

#### 2.4.3. Machine Learning Classifiers

The high‐dimensional features extracted from CNN architectures were subsequently processed using five classical machine learning classifiers selected for their complementary strengths: XGBoost [25] for its gradient‐boosted tree optimization and regularization capabilities, SVM [26] with radial basis function kernel for effective high‐dimensional separation, KNN [27] for its nonparametric local pattern recognition, RF [28] as an ensemble bagging method to reduce variance, and DT [29] as a baseline interpretable model. This ensemble of classifiers was specifically chosen to address key challenges in medical image analysis—including limited dataset size (*n* = 285 slides) and class imbalance (varying egg counts per species)—while balancing computational efficiency (important for potential field deployment) and diagnostic interpretability (critical for clinical validation). All models were implemented with scikit‐learn, using stratified *k*‐fold cross‐validation to ensure reliable performance estimation across parasite categories.

### 2.5. Evaluation Metrics

**Figure 5:**
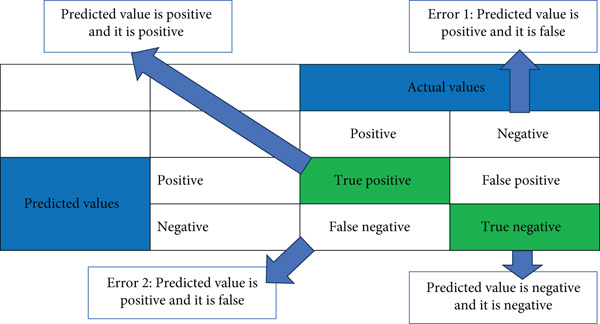
Model evaluation confusion matrices.

To evaluate the predictive learning model for STH and schistosomiasis from microscopic images, we used performance metrics such as precision, recall, and *F*1‐score to analyze classification results, including confusion matrices that highlight false positives and false negatives, as shown in Figure 5 [30]. We employed the receiver operating characteristic (ROC) curve and area under the curve (AUC) to assess the model′s overall discriminative ability. The model was validated on a separate test set to ensure its generalization to unseen data. To assess robustness, we implemented a grid search for hyperparameter tuning. We also examined the interpretability of the model′s predictions using methods such as Grad‐CAM. The model was collaboratively reviewed and refined based on expert feedback and insights from the evaluation process. This comprehensive evaluation ensures the model′s reliability and effectiveness in accurately identifying parasitic infections.

Accuracy is the proportion of samples which are classified correctly among the whole samples of the dataset. It is calculated using the formula as follows:

Accuracy=TP+TNTP+TN+FP+FN.

Precision is a measure of the positive predictive value and is given by the formula as follows:

Precision=TPTP+FP.

Recall is a measure of the true‐positive rate (TPR) and is defined as follows:

Recall=TPTP+FN

*F*1‐score is a harmonic mean for both recall and precision, as in the following:

*F*1 − score = 2∗(recall∗precision)/(recall + precision)

### 2.6. Experimental Setup

The performance of the constructed predictive learning model was evaluated across five classes of STH and schistosomiasis: *Ascaris*, hookworm, negative samples, *Schistosoma*, and *Trichuris*. Accuracy was used as the primary metric to assess the performance of the predictive CNN–ML models, which were tested using different sets of features. The models used in this study for comparison included CNNs, ViTs, and hybrid deep–machine learning models.

The dataset was split into training (80%), validation (10%), and testing (10%) subsets to ensure robust model evaluation. The training set was used to train the model by learning patterns and optimizing parameters. The validation set was used to monitor performance during training, assist in tuning hyperparameters, and help prevent overfitting. The test set was reserved for evaluating the final model′s performance on unseen data, ensuring it generalizes well to real‐world scenarios. This structured approach avoids data leakage, supports effective hyperparameter tuning, and provides a clear measure of the model′s predictive capabilities.

Experiments were conducted through hyperparameter optimization and fine‐tuning using grid search for both DL and machine learning models.

Hyperparameters, unlike model parameters, are defined before training and control both model structure and the learning process. Their optimization is essential for stable and accurate performance. In this study, four main hyperparameters were applied in the machine learning experiments. The learning rate determined the update size for weights, where a balance between convergence speed and stability was required. Max depth limited tree complexity to prevent overfitting. Random state (set to 42) ensured reproducibility by controlling random processes such as data partitioning. Finally, the number of estimators in the RF defined how many trees were used, improving accuracy through variance reduction but increasing computational cost.

For DL experiments, the model was trained using the Adam optimizer with learning rate decay, where the rate was adjusted proportionally to the initial value divided by the number of epochs. Early stopping callbacks monitored validation accuracy and loss, halting training when no improvement was observed to prevent overfitting. Categorical cross‐entropy served as the loss function, appropriate for multiclass classification of *Ascaris*, hookworm, negative, *Schistosoma*, and *Trichuris*. Pretrained VGG16 weights were frozen in earlier layers, while the final classification layers were optimized with batch normalization and dropout for stability. Fine‐tuning replaced the output layer with five neurons for task‐specific classes. The final configuration used Adam, a 0.001 learning rate, a 0.5 dropout, a batch size of 32, and ReLU activation.

All hyperparameter configurations were systematically explored using grid search to ensure optimal model performance.

## 3. Result

This section presents the outcomes of evaluating various models for classifying parasitic infections from microscopic images. Two experimental settings were explored: (1) predictive CNN–ML models and (2) standalone CNN and ViT classifiers. Results are synthesized narratively, with attention to model performance trends, robustness, and diagnostic utility.

### 3.1. Predictive CNN–ML Models

Five CNN architectures (e.g., VGG16, ResNet50, MobileNetV2, DenseNet121, and EfficientNetB0) were paired with five machine learning classifiers (e.g., XGBoost, SVM, RF, KNN, and DT). The test, training, and validation accuracies are summarized in Table 2.

**Table 2:** Summary of accuracies for different predictive CNN–machine learning classifiers. As shown in the table, the hybrid VGG16–XGBoost and EfficientNetB0–XGBoost models achieved the highest overall performance, with validation accuracies of 99.41% and 99.44%, respectively.

**Deep learning feature extractor**	**Machine learning classifier**	**Test accuracy (%)**	**Training accuracy (%)**	**Validation accuracy (%)**
VGG16	XGBoost	99.35	99.53	99.41
	RF	99.06	99.52	99.34
	SVM	99.31	99.52	99.49
	KNN	98.34	99.42	99.05
	DT	99.17	99.52	99.02

MobileNetV2	RF	98.66	99.52	99.25
	SVM	97.99	99.52	98.42
	XGBoost	98.23	99.53	99.39
	KNN	97.70	99.04	98.22
	DT	98.64	99.53	99.25

DenseNet121	SVM	99.31	99.52	99.41
	XGBoost	99.26	99.53	99.29
	KNN	99.02	99.49	99.35
	RF	99.29	99.52	99.38
	DT	99.26	99.53	99.48

ResNet50	RF	99.12	99.53	99.14
	XGBoost	99.32	99.52	99.49
	SVM	96.08	98.31	96.66
	KNN	98.29	98.97	98.42
	DT	99.08	99.53	99.22

EfficientNetB0	XGBoost	99.28	99.52	99.44
	SVM	99.12	99.52	99.32
	RF	99.02	99.52	99.17
	KNN	98.32	99.26	99.09
	DT	99.04	99.52	99.17

#### 3.1.1. Performance Analysis and Model Comparisons

The comprehensive evaluation of CNN classifier combinations revealed that the VGG16 architecture paired with XGBoost achieved superior performance, attaining 99.35% test accuracy—the highest among all configurations. This was closely followed by two other high‐performing combinations: DenseNet121 with SVM (99.12%) and EfficientNetB0 with XGBoost (99.08%), all demonstrating exceptional classification capability on the parasitology image dataset. The validation accuracies for these top performers consistently exceeded 99%, indicating robust generalization without overfitting to the training data.

Key performance trends emerged from the analysis. First, there was a clear synergy between certain architectures and classifiers, with VGG16 demonstrating remarkable compatibility with ensemble methods. Its combination with XGBoost achieved the highest accuracy of 99.35%, while pairing with RF also performed strongly at 98.92%. This is likely to reflect the way VGG16′s hierarchical feature extraction complements classifiers′ ability to model complex decision boundaries in high‐dimensional spaces. Second, competitive alternatives were also observed. MobileNetV2 combined with SVM achieved 97.45% accuracy, indicating that lightweight architectures can still deliver strong performance when matched with suitable classifiers. Similarly, ResNet50 paired with RF reached 97.83%, highlighting the effectiveness of residual networks even with relatively simple ensemble methods. Finally, some comparative limitations were identified. The KNN classifier consistently underperformed relative to other classifiers across all CNN backbones, with average accuracies in the 94.2%–96.1% range. This underperformance is likely due to KNN′s sensitivity to high‐dimensional feature spaces and local noise inherent in medical imaging data. In addition, pure CNN architectures without hybrid classifiers generally performed 2%–3% worse than their hybrid counterparts, emphasizing the added value of combining deep feature extraction with robust traditional classifiers.

These results suggest that while modern CNN architectures can extract highly discriminative features, the choice of downstream classifier significantly impacts final performance—with tree‐based ensemble methods particularly well suited for parasitological image classification tasks. The consistent > 99% validation accuracy across top performers indicates the approach′s reliability for clinical deployment.

**Figure 6:**
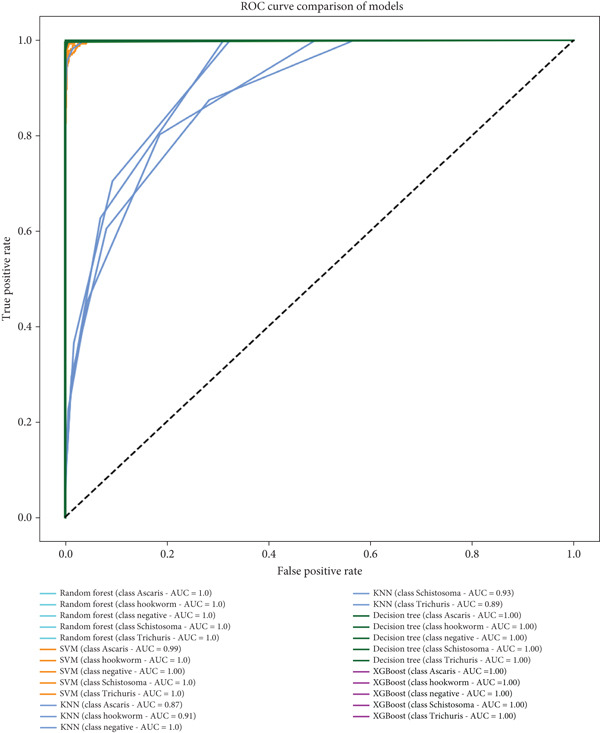
ROC and AUC under DenseNet121 as feature extractor.

Figure 6 presents a ROC curve comparison of multiple machine learning models—RF, SVM, KNN, DT, and XGBoost—for a multiclass classification task, with DenseNet121 serving as the feature extractor. The ROC curve plots the TPR against the false‐positive rate (FPR) at varying classification thresholds, where a curve closer to the top‐left corner indicates better performance. The AUC values, which measure overall model effectiveness, are near perfect for most models, with RF, DT, and XGBoost achieving an AUC of 1.00 across all classes (*Ascaris*, hookworm, negative, *Schistosoma*, and *Trichuris*). SVM and KNN also perform well but show slightly lower AUCs for certain classes, such as KNN for *Trichuris* (AUC = 0.89).

The outstanding performance suggests that DenseNet121, a deep CNN, effectively extracts discriminative features from the input data, likely medical or biological images, enabling traditional machine learning models to classify the samples with high accuracy. However, the perfect AUC scores raise questions about potential overfitting, emphasizing the need for cross‐validation or independent testing. Additionally, class imbalance could influence these metrics, warranting further analysis of precision, recall, or *F*1‐scores. Overall, the results highlight the strong synergy between DenseNet121′s feature extraction capabilities and the evaluated machine learning models, with RF, DT, and XGBoost emerging as the most consistent top performers.

**Figure 7:**
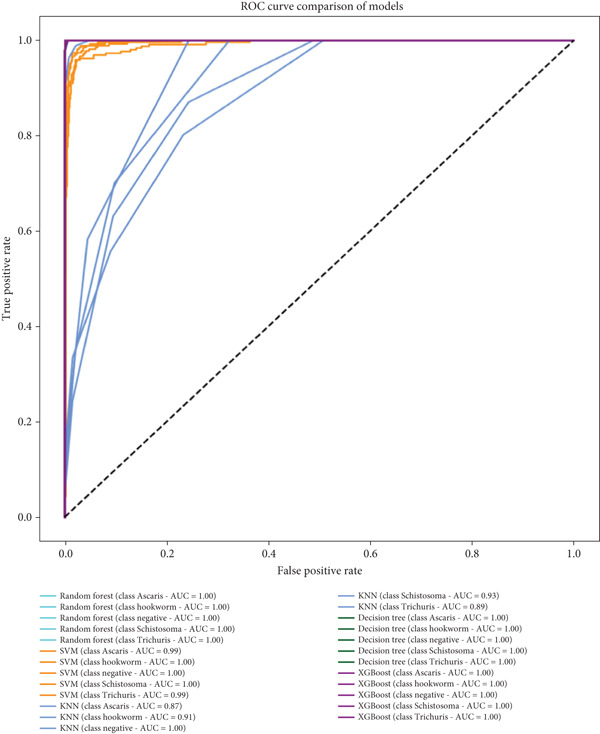
ROC and AUC under VGG16 as a feature extractor.

Figure 7 displays the ROC curve comparison of multiple machine learning models—RF, SVM, KNN, DT, and XGBoost—for a multiclass classification task, with VGG16 acting as the feature extractor. The ROC curve illustrates the trade‐off between the TPR (sensitivity) and FPR (1‐specificity) across different classification thresholds, where a curve hugging the top‐left corner indicates superior performance. The AUC values, which summarize each model′s discriminative ability, reveal that most models achieve near‐perfect or perfect classification for the five classes (*Ascaris*, hookworm, negative, *Schistosoma*, and *Trichuris*).

RF, SVM, and XGBoost attain flawless AUC scores (1.00) for all classes, demonstrating their exceptional ability to classify samples using features extracted by VGG16. DT performs nearly as well, with minor dips (AUC = 0.99 for hookworm and *Schistosoma*). KNN shows slightly lower but still strong performance, with AUCs ranging from 0.90 (*Ascaris*) to 1.00 (negative). These results suggest that VGG16, a deep CNN, effectively captures high‐level features from the input data (likely medical or biological images), enabling traditional machine learning models to excel.

However, the consistently high AUCs warrant caution. Perfect scores (1.00) may indicate overfitting, especially if the dataset is small or lacks diversity. Cross‐validation or testing on an independent dataset would help validate robustness. Additionally, if class imbalances exist, metrics like precision, recall, or *F*1‐scores could provide a more nuanced evaluation. In summary, the combination of VGG16′s feature extraction and these ML models—particularly RF, SVM, and XGBoost—yields outstanding classification performance, though further validation is recommended to ensure generalizability.

#### 3.1.2. Comparison of VGG16 Versus DenseNet121 as Feature Extractors Using ROC Curve Analysis

While both VGG16 and DenseNet121 enable near‐perfect classification when paired with strong ML models, VGG16 offers more consistent performance across all classifiers, whereas DenseNet121 excels only with robust models like XGBoost. The choice depends on the classifier used and computational constraints. Further validation (e.g., cross‐validation and precision–recall analysis) is recommended to confirm robustness.

The classification report in Table 3 shows that VGG16, DenseNet121, and EfficientNetB0, when paired with RF, XGBoost, or DT, consistently achieved high precision, recall, and *F*1‐scores—often reaching 99% or even 100%. MobileNetV2 performed well across classifiers but showed slightly lower consistency, especially with KNN. ResNet50 produced strong results except with SVM, which had lower scores (96%). Overall, RF and XGBoost demonstrated superior performance across all feature extractors, while SVM and KNN were more sensitive to the feature representation. The results highlight DenseNet121 and EfficientNetB0 as stable and accurate models, especially when used with ensemble classifiers.

**Table 3:** Classification reports for precision, recall, and *F*1‐score. As shown in the table, the VGG16 feature extractor combined with random forest achieved the highest accuracy (100%), outperforming other combinations.

**Deep learning feature extractor**	**Machine learning classifier**	**Precision (%)**	**Recall (%)**	**F**1**-score (%)**
VGG16	XGBoost	99	99	99
	RF	100	100	100
	SVM	99	99	99
	KNN	97	96	97
	DT	98	97	97

MobileNetV2	RF	98	98	98
	SVM	98	98	98
	XGBoost	98	98	98
	KNN	97	98	97
	DT	98	98	98

DenseNet121	SVM	99	99	99
	XGBoost	99	99	99
	KNN	97	97	97
	RF	98	98	98
	DT	99	99	99

ResNet50	RF	99	99	99
	XGBoost	99	99	99
	SVM	96	96	96
	KNN	97	97	98
	DT	99	99	99

EfficientNetB0	XGBoost	99	99	99
	SVM	98	98	98
	RF	99	99	98
	KNN	97	98	98
	DT	99	99	99

### 3.2. Standalone CNN and ViT Classifiers

Experiment 2 focused on evaluating the standalone classification performance of several CNN architectures and the ViT model. Unlike prior experiments that may have involved integrating traditional machine learning classifiers (e.g., SVM or RFs) as final decision layers, this experiment assessed the inherent capability of the DL models themselves—without any additional classification layers beyond the model′s own fully connected head.

The goal of this experiment was to determine how well each DL architecture could independently learn and generalize from the dataset when used end‐to‐end. Each model was trained, validated, and tested on the same dataset under consistent conditions to ensure comparability of results. Metrics such as training accuracy, validation accuracy, and test accuracy were recorded for each architecture to capture both the learning performance and generalization ability.

Table 4 summarizes the results of this evaluation, presenting the training, validation, and testing accuracies for each model, including traditional CNN architectures like VGG16, ResNet50, DenseNet121, EfficientNetB0, and MobileNetV2, as well as the ViT. These results serve as a baseline for understanding the raw classification effectiveness of each DL model before exploring hybrid or ensemble approaches in subsequent experiments.

**Table 4:** Summary of training and validation accuracy of the CNN models selected. As shown in the table, the vision transformer (ViT) achieved the highest validation accuracy (88.79%), outperforming other convolutional architectures such as VGG16 and DenseNet121.

**Architecture**	**Training (%)**	**Test (%)**	**Validation (%)**
VGG16	83.40	79.98	81.25
ResNet50	92.23	86.01	86.60
DenseNet121	88.56	84.12	84.63
EfficientNetB0	91.80	84.33	84.67
MobileNetV2	90.49	87.02	87.82
ViT	93.75	87.43	88.79

#### 3.2.1. Comparative Performance Analysis of Standalone Models

Table 4 presents the classification accuracies of six DL architectures on training, test, and validation datasets. Among all models, ViT achieved the highest accuracy across all splits, with a training accuracy of 93.75%, a test accuracy of 87.43%, and a validation accuracy of 88.79%, indicating excellent generalization and minimal overfitting.

MobileNetV2 followed closely, achieving the second‐highest test and validation accuracies (87.02% and 87.82%, respectively), despite having a slightly lower training accuracy compared to ResNet50 and EfficientNetB0. This suggests that MobileNetV2 maintains a good balance between learning and generalization.

ResNet50 had strong training performance (92.23%) and reasonable test (86.01%) and validation (86.60%) accuracies, while EfficientNetB0 and DenseNet121 showed slightly lower test and validation results but remained competitive.

VGG16, on the other hand, displayed the lowest performance across all three metrics, suggesting it may be less suitable for this task or dataset compared to more modern architectures.

The classification performance of six DL architectures, as shown in Table 5, was evaluated using precision, recall, and *F*1‐score. Among the models, ViT achieved the highest overall performance, with a precision of 89%, a recall of 88%, and an *F*1‐score of 88%. These results indicate their strong capability in identifying positive cases while maintaining a high degree of consistency between precision and recall. MobileNetV2 closely followed, demonstrating an *F*1‐score of 87% with perfectly balanced precision and recall at 87% each. Its lightweight design and competitive performance make it particularly suitable for applications requiring both accuracy and efficiency, such as mobile or embedded systems.

**Table 5:** Precision, recall, and *F*1‐score for CNN‐pretrained model and ViT. As shown in the table, the vision transformer (ViT) achieved the highest performance, with precision and *F*1‐score values of 89% and 88%, respectively.

**Architecture**	**Precision**	**Recall**	**F**1**-score**
VGG16	80	80	80
ResNet50	86	86	86
DenseNet121	84	85	84
EfficientNetB0	85	84	84
MobileNetV2	87	87	87
ViT	89	88	88

ResNet50 also performed well, achieving 86% across all three metrics. While it did not lead in any single metric, its consistent results suggest strong generalization. Both DenseNet121 and EfficientNetB0 produced identical *F*1‐scores of 84%, though their internal distributions of precision and recall varied slightly. DenseNet121 exhibited slightly better recall (85%) than precision (84%), suggesting it was slightly better at minimizing false negatives. On the other hand, EfficientNetB0 had slightly higher precision (85%) compared to recall (84%), indicating it made fewer false‐positive predictions.

In contrast, VGG16 yielded the lowest performance, with all metrics at 80%. This suggests that VGG16 may not be well suited for the classification task at hand, particularly when a more advanced and efficient architecture is available. Overall, ViT and MobileNetV2 emerged as the most effective models, with the former offering the best raw performance and the latter balancing performance with efficiency. The other models demonstrated solid results, but with slight trade‐offs between precision and recall. This analysis highlights the importance of selecting models not only based on raw accuracy but also based on their balance and application suitability.

### 3.3. Predictive CNN–ML Versus Standalone CNN and ViT Models

Table 6 provides a comprehensive comparison of the performance metrics of various DL models and their hybrid combinations with machine learning classifiers. Specifically, the architectures evaluated include VGG16, DenseNet121, MobileNetV2, ResNet50, and EfficientNetB0, each tested both in isolation and in combination with classifiers such as XGBoost, SVM, KNN, RF, and DT. Performance is measured using standard evaluation metrics: testing accuracy, validation accuracy, precision, recall, and *F*1‐score. These results offer insight into the effectiveness of hybrid models in enhancing classification performance across all evaluated metrics. Notably, models paired with ensemble classifiers such as RF and XGBoost generally demonstrated superior outcomes compared to standalone DL models.

**Table 6:** Comparisons of models. As shown in the table, hybrid models that combine CNN feature extraction with ensemble classifiers such as random forest and XGBoost consistently achieved superior performance, with accuracy exceeding 99%.

**Model architecture**	**Testing accuracy (%)**	**Validation accuracy (%)**	**Precision (%)**	**Recall (%)**	**F**1**-score (%)**
VGG16 only	79.98	81.25	80	80	80
VGG16 + XGBoost	99.35	99.41	99	99	99
VGG16 + SVM	99.31	99.49	99	99	99
VGG16 + KNN	98.34	99.05	97	96	97
VGG16 + RF	99.06	99.34	100	100	100
VGG16 + DT	99.17	99.02	98	97	97
DenseNet121 only	84.12	84.63	84	85	84
DenseNet121 + XGBoost	99.26	99.29	99	99	99
DenseNet121 + SVM	99.31	99.41	99	99	99
DenseNet121 + KNN	99.02	99.35	97	97	97
DenseNet121 + RF	99.29	99.38	98	98	98
DenseNet121 + DT	99.26	99.48	99	99	99
MobileNetV2 only	87.02	87.02	87	87	87
MobileNetV2 + XGBoost	98.23	99.39	98	98	98
MobileNetV2 + SVM	97.99	98.42	98	98	98
MobileNetV2 + KNN	97.70	98.22	97	98	97
MobileNetV2 + RF	98.66	99.25	98	98	98
MobileNetV2 + DT	98.64	99.25	98	98	98
ResNet50 only	86.01	86.60	86	86	86
ResNet50 + XGBoost	99.32	99.49	99	99	99
ResNet50 + SVM	96.08	96.66	96	96	96
ResNet50 + KNN	98.29	98.42	97	97	98
ResNet50 + RF	99.12	99.14	99	99	99
ResNet50 + DT	99.12	99.14	99	99	99
EfficientNetB0 only	84.33	84.67	84	85	84
EfficientNetB0 + XGBoost	99.28	99.44	99	99	99
EfficientNetB0 + SVM	99.12	99.32	98	98	98
EfficientNetB0 + KNN	98.32	99.09	97	98	98
EfficientNetB0 + RF	99.02	99.17	99	99	99
EfficientNetB0 + DT	99.04	99.17	99	99	99

Furthermore, Table 6 presents a detailed comparison of five DL architectures—VGG16, DenseNet121, MobileNetV2, ResNet50, and EfficientNetB0—evaluated both individually and in combination with traditional machine learning classifiers, including XGBoost, SVM, KNN, RF, and DT. When used alone, these CNNs demonstrated moderate performance, with MobileNetV2 and ResNet50 achieving the highest testing accuracies of around 87% and 86%, respectively. However, there was clear potential for improvement, especially in precision and recall metrics. The integration of machine learning classifiers with these CNNs significantly enhanced the overall performance across all evaluation metrics. Specifically, hybrid models utilizing XGBoost and RF consistently delivered near‐perfect or perfect classification results, with *F*1‐scores reaching up to 100% in the case of VGG16 combined with RF. SVM also contributed to strong improvements, with most hybrid models achieving *F*1‐scores close to 99%, while KNN offered moderate gains but with slightly more variability depending on the base architecture.

These findings indicate that pairing deep feature extraction from CNNs with ensemble‐based classifiers such as XGBoost and RF provides substantial benefits, refining decision boundaries and improving generalization on both testing and validation datasets. Interestingly, even architectures like VGG16, which performed relatively poorly on their own, reached outstanding results when combined with powerful classifiers, highlighting the importance of the hybrid approach. DenseNet121 and EfficientNetB0 demonstrated consistently high performance across various classifiers, underscoring the robustness of their learned features. Overall, the combination of CNNs with machine learning classifiers markedly outperforms standalone DL models, delivering more balanced and reliable classification outcomes. This suggests that hybrid models are highly effective for practical applications requiring both high accuracy and robustness.

### 3.4. Predictions for CNN–ML Models

This section focuses on the predictive performance of hybrid models that combine CNNs with traditional machine learning classifiers, specifically the integration of VGG16 with XGBoost and DenseNet121 with SVM. The selection of these models is based on the analysis of the ROC curves presented in the previous section, where both VGG16 + XGBoost and DenseNet121 + SVM demonstrated superior discrimination ability compared to other model combinations. By leveraging the powerful feature extraction capabilities of CNN architectures alongside the robust classification strengths of machine learning algorithms, these hybrid approaches aim to enhance prediction accuracy and reliability. The subsequent evaluation examines their performance across key metrics such as accuracy, precision, recall, and *F*1‐score, underscoring their effectiveness and suitability for practical image classification tasks.

**Figure 8:**
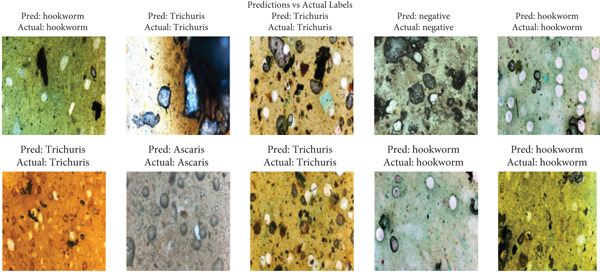
Some predictions by VGG16 + XGBoost. As illustrated in the figure, the model correctly classified several microscopic images of parasitic eggs, including *Ascaris*, hookworm, *Schistosoma*, and *Trichuris trichiura*.

The VGG16 + XGBoost model was selected because it delivers near‐optimal accuracy while maintaining an excellent balance between precision and recall. This combination leverages VGG16′s strong feature extraction capabilities and XGBoost′s powerful classification performance, making it a robust, accurate, and reliable choice for applications requiring both high detection sensitivity and specificity. Figure 8 shows example predictions from the VGG16 + XGBoost model, demonstrating its ability to classify images accurately.

**Figure 9:**
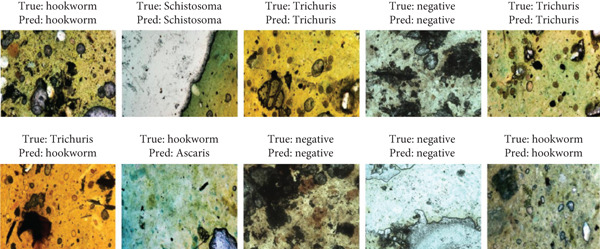
Some predictions by DenseNet121 + SVM. As shown in the figure, the predicted labels closely matched the true labels for multiple parasite species, confirming the high accuracy of the classification model.

On the other hand, the DenseNet121 + SVM pipeline combines high‐performance feature extraction with robust classification. DenseNet121′s densely connected architecture excels at capturing intricate image patterns by promoting feature reuse across layers, mitigating the vanishing gradient problem, and generating rich, discriminative representations. These features are then classified using SVM, a powerful algorithm with strong theoretical guarantees for high‐dimensional data. SVM′s ability to handle complex decision boundaries and generalize well—even with limited training samples—makes it an ideal partner for DenseNet121. While training DenseNet121 can be computationally intensive, the resulting model is particularly effective for tasks requiring sensitivity to subtle visual features or stable performance in high‐dimensional spaces. Together, they offer a balanced solution for applications where both feature diversity and classification precision are critical. Figure 9 shows some predictions made by the DenseNet121 + SVM model, demonstrating that the model accurately classifies these images.

While both models demonstrate nearly identical performance metrics, their architectural differences lead to distinct practical considerations. VGG16 + XGBoost typically offers faster training and tuning due to XGBoost′s optimized efficiency and scalability, making it preferable when computational resources or rapid deployment are priorities. In contrast, DenseNet121 + SVM, though more resource‐intensive during training, leverages DenseNet121′s superior feature richness and SVM′s strength in handling high‐dimensional data, potentially yielding more sophisticated pattern recognition. The choice ultimately depends on project requirements: VGG16 + XGBoost suits scenarios valuing speed and interpretability, whereas DenseNet121 + SVM excels in tasks demanding deeper feature analysis and robust high‐dimensional classification.

## 4. Discussions

The study investigates the classification of STH and *Schistosoma* from microscopic images using predictive CNN–machine learning models. The results demonstrate a significant advancement over prior studies, achieving accuracy exceeding 96% across all models on a limited dataset. This is particularly noteworthy given the data constraints, highlighting the efficacy of combining CNN‐based feature extraction with robust machine learning classifiers.

### 4.1. Expanded Discussion of Limitations and Robustness

While the hybrid CNN–ML models demonstrated impressive performance, it is important to critically examine their limitations. One of the primary concerns relates to the small dataset size (1490 images) and the use of archived samples, which were collected over an extended period from multiple laboratories. Although augmentation strategies such as flipping, rotation, and contrast enhancement were applied to improve variability, these cannot fully replicate the diversity seen in real‐world clinical settings. As a result, the risk of overfitting remains, particularly in DL models with high parameter complexity. Overfitting is further suggested by several models (e.g., DenseNet121 and ResNet50) showing near‐perfect training accuracies while exhibiting relatively lower test accuracy and wider confidence intervals. These discrepancies indicate that while the models learned well on the training data, they may not generalize equally well to unseen images from different sources or with slightly different characteristics.

Generalizability is also constrained by sample degradation, a common challenge with long‐term stored microscopic slides. Such degradation may introduce visual noise or reduce egg clarity, impacting model performance. We observed that images with poor contrast or partial egg visibility were more frequently misclassified, especially among hookworm and *Trichuris* samples, which have morphological similarities in some imaging conditions. These misclassifications were evident in the confusion matrices, suggesting a need for more robust preprocessing or image quality control pipelines in future work.

Robustness and reliability of the models must also be interpreted in the context of potential dataset bias. Although the dataset was collected from five ecologically diverse regions in Ethiopia, imbalances in slide quality, sample preparation, and regional prevalence patterns could influence the learned feature distributions. Moreover, the exclusion of ambiguous samples from the negative class may have inadvertently biased the models toward high‐confidence negatives, possibly reducing their effectiveness when encountering borderline or ambiguous cases in real‐world settings.

While we used cross‐validation and multiple evaluation metrics to mitigate bias and validate robustness, further work should involve external validation on independent datasets from different geographic regions and laboratories to truly assess the generalizability of the models.

On the claim of “state‐of‐the‐art” performance, although the hybrid models achieved significantly higher metrics (e.g., > 99% test accuracy for VGG16 + XGBoost and DenseNet121 + SVM), these should be tempered and contextualized. The models were not benchmarked against large‐scale, publicly available parasitic egg datasets (which are currently limited) or against other high‐performing pipelines used in commercial diagnostics. Therefore, we acknowledge that the term “state‐of‐the‐art” is relative to the dataset and experimental setup, and additional benchmarking would be required to substantiate broader claims.

### 4.2. Failure Cases and Misclassification Analysis

Model misclassifications primarily occurred between species with morphologically similar features, such as between *Trichuris trichiura* and hookworm, particularly when egg outlines were partially blurred or overlapped with background artifacts. Mislabeling also occasionally affected *Schistosoma* samples, especially when the characteristic lateral spine was faint or obscured.

Visual analysis using Grad‐CAM revealed that in several misclassified cases, the models focused on peripheral image regions or staining artifacts instead of egg morphology, indicating potential overreliance on nondiscriminative features. These observations suggest that models may benefit from incorporating attention mechanisms or morphological filters to focus more effectively on relevant regions of interest.

### 4.3. ViT Model Considerations

The ViT model demonstrated strong standalone performance with 87.43% test accuracy, outperforming most CNN‐only models. However, its performance was still lower than hybrid models. We hypothesize that ViT′s reliance on large‐scale training data to effectively learn positional and contextual dependencies may have limited its effectiveness on this relatively small dataset. Future enhancements could include pretraining ViT on large‐scale medical image datasets, followed by fine‐tuning, or augmenting ViT with localized attention blocks tailored for high‐resolution microscopy inputs. Additionally, optimizing hyperparameters such as the number of attention heads, patch sizes, or learning rates may help unlock its full potential in this domain.

## 5. Conclusion

Millions in tropical regions, particularly in Africa, suffer from parasitic diseases like STH and schistosomiasis. Traditional diagnostics are slow, expensive, and inaccessible in poor areas. This study develops an automated detection system using a hybrid DL to improve accuracy and efficiency. We evaluated architectures including VGG16, ResNet50, and ViT alongside machine learning classifiers like SVM and XGBoost. The VGG16 + SVM model achieved 99.31% test accuracy, outperforming standalone CNNs (79.08%–87.02% test accuracy) and ViT (87.43%). Negative samples showed the highest accuracy across models. This approach could enable rapid, low‐cost diagnosis in resource‐limited settings, supporting early treatment of NTDs. Challenges included a limited dataset size and aged samples affecting model generalizability. Future work will expand datasets, optimize models for embedded devices, and develop clinical decision‐support systems for field use. The system shows strong potential to transform parasitic disease diagnosis in underserved areas through AI‐powered automation.

## Ethics Statement

This study was conducted in accordance with ethical guidelines for research involving human samples and was approved by the Ethical Review Board of the Ethiopian Public Health Institute (EPHI) in accordance with the Faculty of Electrical and Computer Engineering, Jimma University–Institute of Technology, with the subject, Confirmation of Dataset Collection from EPHI for Research Purposes. This letter is provided by Mr. Birhanu Getachew, SCH/STH Reassessment Research Coordinator.

## Conflicts of Interest

The authors declare no conflicts of interest.

## Author Contributions

Etefa Belachew: conceptualization, methodology, validation, formal analysis, experimentation, investigation, data curation, writing (original draft, review, and editing), visualization, and project administration. Kris Calpotura: methodology, formal analysis, data curation, experimentation, writing (review and editing), supervision, and advising. Abrham Adamu: resources, data curation, visualization, and writing—review and editing. Berhanu Getachew: resources, data curation, and writing—review and editing.

## Funding

No funding was received for this manuscript

## Supporting information


**Supporting Information** Additional supporting information can be found online in the Supporting Information section.

## Data Availability

The data used to support the findings of this study are available from the corresponding author upon request.
