# Supplementary figures and images for: Constructing a Predictive Model for STH and Schistosomiasis Classification From Microscopic Images
Source: Biomed Res Int. 2025 Nov 29;2025:8074581. doi: 10.1155/bmri/8074581 (PMC12663861; doi:10.1155/bmri/8074581)

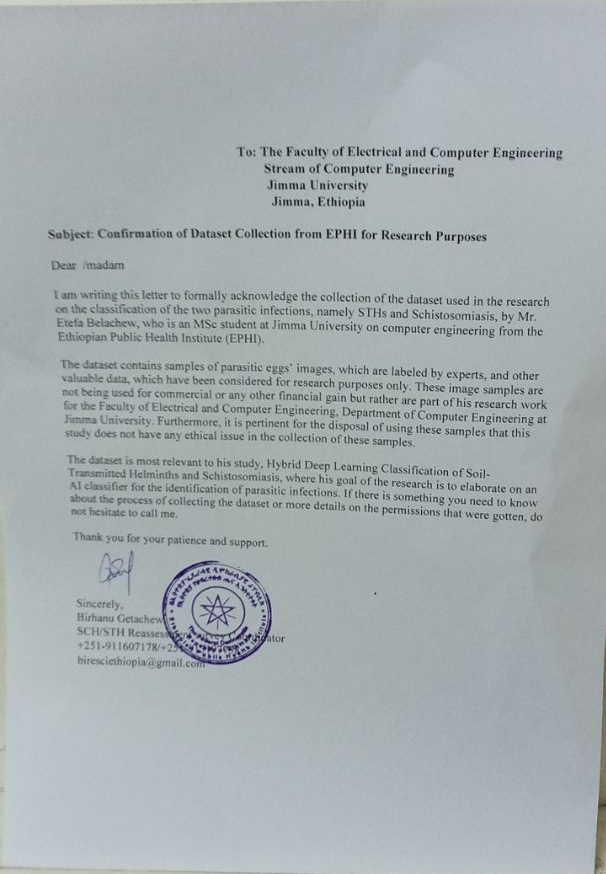

Supplement: Supplementary file 1 — Supporting Information Additional supporting information can be found online in the Supporting Information section. [file BMRI-2025-8074581-s001.png]
